# Primary adenocarcinoma of the renal pelvis: An uncommon tumor, a case report

**DOI:** 10.1016/j.ijscr.2024.109887

**Published:** 2024-06-06

**Authors:** Wael Gazzah, Mahdy Mzoughi, Rayen Lahouar, Badreddine Benkhalifa, Bacem Zaidi, Braiek Salem

**Affiliations:** a University of Sousse, faculty of Medicine, Department of Urology, Ibn El Jazzar Hospital, Kairouan, Tunisia; b University of Sousse, Faculty of Medicine, Department of Surgery, Ibn El Jazzar Hospital, Kairouan, Tunisia

**Keywords:** Adenocarcinoma, Renal pelvis neoplasms, Nephroureterectomy, Diagnostic imaging, Urology

## Abstract

**Introduction and importance:**

Primary adenocarcinoma of the renal pelvis is a rare and unique malignancy, representing a small fraction of renal cancers and posing significant diagnostic challenges due to its unusual presentation and similarity in symptoms to more common excretory tract disorders. This case emphasizes the importance of distinguishing this pathology from other renal neoplasms and metastatic adenocarcinomas that originate in the digestive tract.

**Case presentation:**

We report the case of a 34-year-old man with no significant medical history who presented persistent lower back pain but no hematuria, which is atypical for renal pathologies. Initial imaging identified a 30 × 14 mm enhancement mass in the right renal pelvis. Surgical intervention was performed through right nephroureterectomy, including excision of the bladder cuff. Histopathological examination confirmed the diagnosis of primary intestinal-type adenocarcinoma of the renal pelvis, characterized by necrotic carcinomatous proliferation with varying architectural patterns and occasional signet ring cells.

**Clinical discussion:**

The diagnosis of primary renal pelvis adenocarcinoma is complicated by its nonspecific symptomatology and the potential for misdiagnosis as a more common urothelial carcinoma or a metastatic digestive-origin adenocarcinoma. Immunohistochemical staining supported a primary rather than metastatic digestive tract origin. This case underscores the need for a comprehensive diagnostic approach, including advanced imaging and meticulous histopathological analysis, to effectively differentiate this rare entity from other neoplasms.

**Conclusions:**

This case highlights the diagnostic complexities and the critical need to be aware among clinicians about rare renal cancers such as primary adenocarcinoma of the renal pelvis. It also stresses the importance of interdisciplinary collaboration in the diagnosis and management of such rare cases, improving our understanding and requiring timely and accurate treatment.

## Highlights

• Primary adenocarcinoma of the renal pelvis is a rare and challenging cancer type.
• Symptoms 7often resemble more common urinary tract disorders, complicating diagnosis.
• Treatment involved a right nephroureterectomy and confirmed the diagnosis histologically.
• Diagnostic accuracy requires advanced imaging and thorough pathological examination.
• Effective treatment led to no recurrence at one-year follow-up.

## 1 Introduction

Primary adenocarcinoma of the kidney pelvis is a highly unusual and under-documented cancer, representing a small fraction of urological tumors [1]. Originating uniquely within the renal pelvis, this form of cancer is not only rare, but also distinct in its clinical presentation and pathological characteristics, which differ markedly from the more prevalent urothelial carcinoma [2].

The diagnosis of primary renal pelvis adenocarcinoma is notoriously challenging due to its symptomatology, which often mirrors that of more common disorders of the upper excretory tract. This overlap can mislead clinicians and delay an accurate diagnosis, underscoring the need for increased awareness and precise investigative procedures [3].

To illustrate these complexities, we present the case of a 34-year-old man initially treated for an upper urinary tract tumor. Despite initial interventions, the definitive diagnosis of intestinal-type primary adenocarcinoma of the renal pelvis was only confirmed by a complete histopathological analysis after a total nephroureterectomy. This case exemplifies the critical need for meticulous diagnostic processes to distinguish this rare pathology from more conventional urological conditions. This case report was carried out according to recent SCARE criteria [4].

## 2 Patient and observation

### 2.1 Presentation and diagnosis

**Fig. 1:**
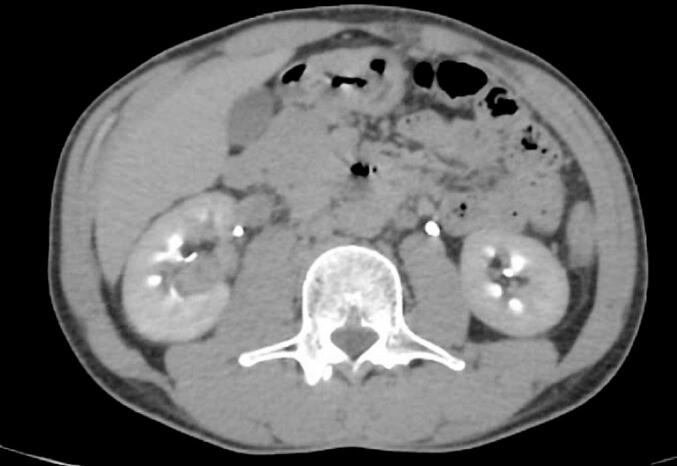
CT revealed a tissue mass measuring 30 × 14 mm in the right renal pelvis.

We report on a 34-year-old man without a significant medical history who had persistent lower back pain lasting several months, especially without hematuria, an atypical presentation of renal pathologies. An initial CT scan (Fig. 1) identified a tissue mass of 30 × 14 mm in the right renal pelvis, showing significant enhancement after iodinated contrast injection, indicative of a vascular neoplasm.

### 2.2 Surgical intervention

Subsequently to the imaging, a right nephroureterectomy was performed, including excision of the bladder cuff. The surgical sample weighed 220 g, comprising a kidney of 11 × 7 × 5 cm, extended by a 21 cm ureter with a diameter ranging from 1 to 2 cm. Upon dissection, a firm grayish-white tumor was observed that spanned 5 cm in its largest dimension within the renal hilum, extending into the perihilar parenchyma and up to the first 4 cm of the ureteral wall.

### 2.3 Pathological examination

**Fig. 2:**
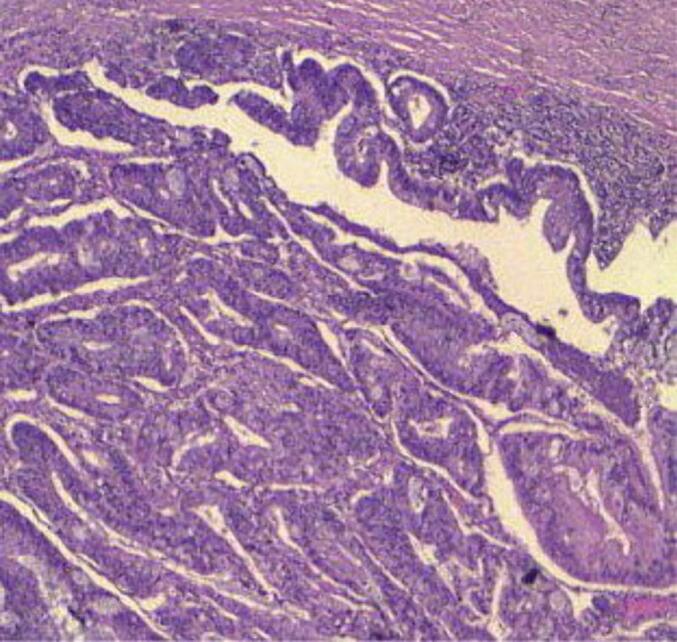
The photomicrograph shows malignant glands and signet-ring cells in large pools of extracellular mucin (Hematoxylin and Eosin.x10).

Microscopic evaluation showed predominantly necrotic carcinomatous proliferation in varying architectural patterns, sheets, groups, cords, and tubules. Tumor cells exhibited basophilic cytoplasm, round to oval nuclei with dusty chromatin, prominent nucleoli, and occasional signet ring cell appearance due to peripheral nucleation by intracytoplasmic mucus (Fig. 2). Notable were carcinomatous endovascular emboli and involvement of the perineural sheath.

### 2.4 Immunohistochemical findings

**Fig. 3:**
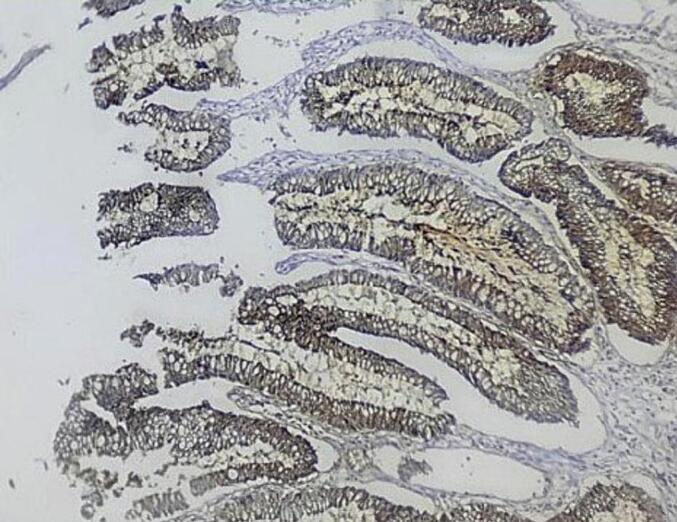
Photomicrograph showing positivity for CK20 (400×).

Immunohistochemical staining was inconsistent, with positive anti-Cyto Keratin (CK)20 and negative anti-CK7, antisynaptophysin, and antichromogranin staining, suggesting the origin of the digestive tract of the adenocarcinoma (Fig. 3). Despite extensive investigations of a primary tumor elsewhere, including comprehensive endoscopic evaluations both upper and lower, all findings were negative, leading to confirmation of the diagnosis of primary intestinal-type adenocarcinoma of the renal pelvis.

### 2.5 Follow-up and outcome

At a 1-year follow-up, the patient did not show symptoms, maintained normal renal function, and showed no signs of recurrence, underscoring the effectiveness of the diagnostic and therapeutic approaches taken.

## 3 Discussion

### 3.1 Histological parallels and rarity

Primary adenocarcinoma of the kidney pelvis is an extremely rare entity, representing <1 % of all tumors of the upper urinary tract, which are predominantly (>95 %) urothelial carcinomas [5]. Ackerman et al. first described this distinct pathology in 1946, and since then fewer than 100 cases have been documented [6]. This rarity underscores the need for increased clinical suspicion and knowledge to diagnose such cases effectively.

### 3.2 Pathogenesis

The pathogenesis of primary adenocarcinoma of the renal pelvis is not well understood, presenting a complex challenge in urologic oncology. Commonly associated with chronic irritation conditions, pyelocaliceal lithiasis often leads to squamous metaplasia, which can progress to squamous cell carcinoma. Similarly, glandular metaplasia may develop under continuous mechanical and inflammatory stress, increasing the risk of developing into dysplasia and eventually adenocarcinoma [7]. Another theory posits that such tumors could arise from the embedding of the urothelial epithelium in the renal parenchyma, a process possibly linked to congenital abnormalities such as horseshoe or poorly formed kidneys [8]. Furthermore, there may be a transitional phase in which adenocarcinoma and in situ urothelial carcinoma coexist, indicating a potential glandular transformation pathway similar to those observed in bladder cancers.

### 3.3 Epidemiology and clinical presentation

The typical discovery of primary renal pelvis adenocarcinoma occurs around the age of 60, making cases before age 35 exceedingly rare, and Gupta et al. report only four such instances [9]. Nonspecific and often misleading symptoms, such as those associated with lithiasis or infection, complicate the diagnosis, as they can obscure the underlying neoplastic process. In addition, standard imaging techniques may not always be definitive, necessitating a high index of suspicion and possibly more targeted diagnostic approaches.

### 3.4 Differential diagnosis challenges

In our case, the primary differential diagnosis was the possibility of metastatic digestive-origin adenocarcinoma in the renal pelvis. This scenario is typically excluded by detailed histopathological and immunohistochemical analyzes. Such distinctions are vital and affect both prognosis and therapeutic strategies. The absence of a prior neoplastic history, coupled with negative endoscopic and radiographic findings, strongly suggests a non-metastatic origin. Although cytokeratin markers (CK7 and CK20) provide limited discriminative power, the use of CDX2 and β-catenin as biomarkers has been explored. The expression of CDX2, although indicative, is not definitive, as it may appear in primary and metastatic adenocarcinomas [10]. In contrast, the absence of β-catenin nuclear staining of -catenin, which is prevalent in approximately 80 % of gastrointestinal-origin adenocarcinomas, supports the diagnosis of a primary tumor when it is not observed.

### 3.5 Treatment and management options

Primary adenocarcinomas are typically aggressive and high-grade. There is no standardized treatment protocol, but early radical surgery is often the preferred initial approach, reflecting the treatment paradigms for common clear cell renal carcinomas. The efficacy of chemotherapy in this context remains uncertain; the M-VAC regimen, typically used for papillary adenocarcinoma, has shown limited success. Alternatively, the TJ protocol, which includes four cycles of paclitaxel and carboplatin, has shown satisfactory results with minimal adverse effects [11].

### 3.6 The importance of early diagnosis

The consequences of delayed diagnosis can be alarming, leading to significant delays in appropriate treatment, which can worsen the patient's prognosis. Given the rarity of this type of cancer and the often nonspecific nature of its symptoms, a high degree of vigilance is crucial when evaluating any kidney mass.

### 3.7 Comprehensive diagnostic approach

A comprehensive diagnostic strategy that integrates advanced imaging techniques and targeted biopsies is essential. However, it is important to note that these techniques are not without complications [12]. Collaborative efforts of an interdisciplinary team, including urologists, radiologists, pathologists, and oncologists, are indispensable. This collaboration ensures not only the precision of the diagnosis, but also the development of an optimal management plan tailored to the unique aspects of each case.

## 4 Conclusions

Primary adenocarcinoma of the renal pelvis represents a formidable diagnostic challenge, attributed to its rarity and the complex nature of its presentation. This case underscores the need to advance our understanding of its pathogenesis and refine diagnostic methodologies to identify this uncommon malignancy with greater precision and speed. Additionally, the development of customized therapeutic strategies remains crucial to improving patient outcomes.

Documentation and dissemination of such rare cases are invaluable, as they enrich the clinical knowledge base and increase awareness within the medical community of this extraordinary pathology. This case not only serves as a reminder of the diagnostic and therapeutic dilemmas posed by such rare conditions, but also highlights the critical need for ongoing research and interdisciplinary collaboration. Future studies should focus on elucidating the molecular mechanisms underlying primary renal pelvic adenocarcinoma and on evaluating the efficacy of various therapeutic protocols in improving long-term survival rates.

By continuing to share insights and updates on rare urological cancers, we can foster a more informed and prepared medical community, ready to address these challenging cases with innovative approaches and robust clinical practices.

## Patient perspective

The patient expressed relief and satisfaction with the treatments received, highlighting their effectiveness and the care provided by the medical team.

## Authors' contributions

All authors contributed equally to this work.

## Informed consent

Written informed consent was obtained from the patient for the publication of this case report and any accompanying images. A copy of the written consent is available for review by the Editor-in-Chief of this journal upon request.

## Source of funding

No funding was received for conducting this study.

## Ethical approval

Ethical approval for this study (Ethical Committee N° RRN 0032/2024) was provided by the Ethical Committee of Ibn El Jazzar Hospital, Kairouan, Tunisia on 29 April 2024.

## Guarantor

Wael Gazzah.

## Declaration of competing interest

The authors declare that they have no competing interest.
